# Flexible Loyalties: How Malleable Are Bicultural Loyalties?

**DOI:** 10.3389/fpsyg.2016.01985

**Published:** 2016-12-20

**Authors:** Andy Y. Chiou, Brittany K. Mercado

**Affiliations:** ^1^Business Management Department, Farmingdale State College (SUNY)Farmingdale, NY, USA; ^2^Department of Management, Baruch College (CUNY)New York, NY, USA

**Keywords:** biculturalism, bicultural identities, social Identities, loyalty conflicts, cultural priming

## Abstract

Biculturals are individuals who are acculturated in two cultures and have dual identities. Due to this, many early discussions on biculturalism argued that biculturals may have divided loyalties between their two cultural backgrounds and the identities derived from these backgrounds. This view is further highlighted given historical and contemporary debate regarding immigrants in the European and American political arenas. These concerns illustrate two possibilities. First, that biculturals have a preference for their home or host culture, identifying one as the in-group to express loyalty toward and the other as the out-group. Second, biculturals may alternate between who they identify as their in-group depending upon the circumstances. In a particular cultural environment, a given bicultural may feel greater degrees of loyalty toward that culture, while feeling different loyalties when immersed in a different cultural environment. To-date, few empirical studies have examined these two questions in detail. We proposed two hypotheses: First, biculturals will express higher levels of loyalty for a specific culture if they have been exposed to a prime congruent with that culture than if they have been exposed to a prime associated with a different culture. Second, the magnitude of preferences expressed for the two cultures will differ depending on the cultural prime. We experimentally investigated this phenomenon in a sample of Chinese-Americans (*N* = 136) using a computer simulated soccer game between the United States and China. This simulation was selected in order to avoid the controversial nature of an immigration or cultural conflict scenario. Past research has shown that support for the sports team of a given country is a form of expressing loyalty. Participants were randomly exposed to one cultural priming condition (American, Neutral, Chinese) using commentaries recorded in different languages: English, no commentary, and Chinese. Participants were then asked to what degree they would cheer for each team. Participants expressed more likelihood to cheer for the Chinese team than for the American team. However, our results indicate that cultural priming does influence the degree to which the participants express loyalty for the Chinese team over the American team in the form of rooting behaviors.

## Introduction

Many early discussions on biculturalism have argued that biculturals, or individuals who have internalized two cultures (Brannen and Thomas, 2010), are sometimes seen as marginal individuals who possess divided loyalties and are ambivalent toward either of their cultural identities (cf. LaFromboise et al., 1993). Although in recent decades research on biculturalism has greatly expanded our understanding of bicultural cognition and identity organization (Hong et al., 2000; Benet-Martínez et al., 2002), while bringing to question the very concept of biculturals as marginal individuals (Pilar and Udasco, 2004), less research has been conducted on the extent to which biculturals identify with either of their cultural backgrounds. This question is of practical importance given issues prevalent in the United States, such as the forced relocation of Japanese-Americans during the Second World War, the anti-Muslim sentiment faced by Muslim- and Arab-Americans (Krieg, 2015), and allegations of espionage faced by Asian-Americans (Purdy, 2001; Asher-Shapiro, 2015; Stewart, 2016) in the twenty first century. That these types of suspicions have so frequently fallen on biculturals in the United States has caused concern and spurred investigation (Asher-Shapiro, 2015).

Despite our current understanding of bicultural cognition and identity, the question of whether biculturals have mixed loyalties still remains. This is critical given increasing cultural complexities not only in the United States, but also in the rest of the world. Biculturals, and by extension multiculturals, will increasingly be placed in positions where they may be seen as needing to choose between their ethnic or the mainstream culture, either at work (e.g., cross-country business negotiations or resource allocation between subordinates) or in their social lives (e.g., support for various political issues). Therefore, to launch investigation into this important issue, we propose the following research question: Given the dual cultural backgrounds that biculturals possess, do they have a preference for their home/ethnic or host/dominant culture, identifying one as the in-group and the other as the out-group when exposed to a specific cultural environment, or do they alternate between who they identify as their in-group depending upon the circumstances?

## Biculturalism

Biculturals are individuals who have gained fluency in two different cultures and internalized both; this fluency provides them with the knowledge of a particular cultural setting necessary to adjust their behavior in accordance with that cultural setting (Mok and Morris, 2009; Mok et al., 2010). These individuals are able to switch between different cultural world views depending on the cultural stimuli they receive (Hong et al., 2000), a phenomenon that extends to the switching of personalities (Ramirez-Esparza et al., 2006; Chen and Bond, 2010).

Bicultural individuals can gain fluency in two cultures through the acculturation process, broadly defined as the “cultural and psychological change that follows intercultural contact” (Berry et al., 2006, p. 305). Individuals who immigrate from one society to another face the challenge of interacting with the host culture while maintaining connections with their home culture. It is this acculturation process that allows biculturals to accumulate cultural knowledge and gradually create cultural identities in two different cultures, allowing them to become bicultural. It should be noted that current research suggests acculturation is not necessarily a unidimensional concept, in which the host culture gradually subsumes the home culture, but instead a bi-dimensional model, in which acculturation in the home and host cultures must be considered separately (cf. Tsai et al., 2000; Nguyen and Benet-Martínez, 2007). Biculturalism has traditionally been viewed as an integrated acculturation strategy (Nguyen and Benet-Martínez, 2007), with consensus in the literature that biculturalism relies on individuals acculturating in and identifying with both home and host cultures. Although an argument may be made that second generation biculturals who are born in a host country differ from first generation biculturals who emigrated from a home country to a host country (Berry and Sam, 1997; Phinney et al., 2001; Liebkind, 2003), current bicultural research has thus far demonstrated that these two groups of biculturals display similarities in terms of cognition (Mok and Morris, 2010; Mok et al., 2010) and identity (Miramontez et al., 2008).

Biculturalism can also occur in other forms, driven primarily by globalization. Arguments have been made that Hong Kong citizens also represent a form of biculturalism due to globalization and British colonization (Bond and Cheung, 1983; Bond, 1993; Hong et al., 2000). While Nguyen and Benet-Martínez (2013) note that Hong Kong biculturals are different due the dominant and heritage cultures being the same, i.e., Asians living in an Asian society, in comparison to immigrant biculturals in which the dominant and heritage cultures are different, e.g., Asians in American society, bicultural priming studies that have succeeded in eliciting different cultural frames (Hong et al., 2000) and identities (Brewer et al., 1987; Ng and Lai, 2009, 2011) in Hong Kong biculturals.

No matter what processes lead to biculturals, whether through immigration or globalization, research has informed us that all biculturals face the need to balance the requirements of their own ethnic culture and the host culture in a constant negotiating act (Nguyen and Benet-Martínez, 2007). This balancing of cultural identities may also be malleable. Extending earlier evidence that biculturals can be primed to see the world through different cultural lenses (cf. Hong et al., 2000), scholars have demonstrated that bicultural identities can be influenced via cultural priming. There are many ways in which culture can be primed, ranging from pronoun circling tasks, scrambled sentence tasks, subliminal tasks (cf. Oyserman and Lee, 2008 for a detailed discussion), to cultural icons (Hong et al., 2000) and language (Ramirez-Esparza et al., 2006; Chen and Bond, 2010). Social identity theory (SIT; Tajfel and Turner, 1979) and self-categorization theory (SCT; Turner et al., 1987), jointly referred to as the social identity approach (SIA), provide insights into influences on the cultural identities of bicultural individuals. Individuals derive a social identity from groups they belong to, leading them to positively evaluate their own group compared to other groups (Tajfel and Turner, 1979). Of equal importance, individuals also possess multiple self-concepts which may be activated depending upon the specific situation; one particular aspect of an individual's self-concept may become more salient in a social context for which said self-concept exhibits the greatest fit (Turner et al., 1987; Hogg et al., 1995). That individuals can recognize multiple in-groups is a phenomenon which has been empirically established (Brewer et al., 1987; Hewstone et al., 1993; Brewer, 1999; Roccas and Brewer, 2002; Kang and Bodenhausen, 2015).

Verkuyten and Pouliasi (2006) further showed that depending on the cultural primes Greek-Dutch biculturals were presented with, they self-identified more as either Greek or Dutch. Greek primes elicited greater self-identification as Greek, while Dutch primes elicited greater self-identification as Dutch (Verkuyten and Pouliasi, 2006). Similar studies have been conducted on Hong Kong biculturals (Ng and Lai, 2011), Mexican-Americans (Devos, 2006), and Chinese-Australians (Liu, 2015), further empirically demonstrating the effects of cultural primes on cultural identification. The priming of individual social identities can also influence decision making, with individuals making decisions that are congruent with the values of the primed identity (LeBoeuf et al., 2010). One possible form in which individuals can engage in such decision making is group loyalty.

Group loyalty in a social identity context can manifest in many different ways, either affective, cognitive, or behavioral (Van Vugt and Hart, 2004). Affective manifestations can relate to the experience of positive emotions through group membership, cognitive manifestations can relate to overall trust in group members, and behavioral manifestations can relate to sacrificing of the self for the betterment of the group (Van Vugt and Hart, 2004). Because acculturation is the process by which individuals gradually identify with given cultures, we can expect biculturals to exhibit various manifestations of group loyalty with the acculturated cultures. Although the aforementioned studies (Devos, 2006; Verkuyten and Pouliasi, 2006; Ng and Lai, 2011; Liu, 2015) have demonstrated that the self-perceived cultural identity of biculturals can be made malleable depending on the cultural context they are exposed to Hogg et al. (1995) review of identity and social identity theory noted the importance of determining how individuals behave toward out-groups in social identity research. As such, little research has been conducted on how biculturals behave toward other individuals or cultural groups given the activation of a cultural identity. Additionally, no studies have yet to place biculturals in a situation where the two cultures they belong to are in direct competition with each other, thereby forcing biculturals to make a direct comparison of loyalties between the two cultural groups when they have been primed with a particular cultural prime.

For biculturals who identify with two different cultures, group loyalty can be complicated. Because they have two possible cultural identities with corresponding in-groups, they could potentially have loyalties toward two cultural groups. This dilemma is amplified in situations when biculturals may be forced to choose which of the two cultural groups to express loyalty toward. However, if the cultural identities of biculturals can be primed, then cultural primes should influence how biculturals express loyalties toward different cultural groups. Biculturals should reflect this malleable identification by expressing increased loyalty for the culture of the primed identity.

*Hypothesis 1*: Biculturals will express higher levels of loyalty for a specific culture if they have been exposed to a prime congruent with that culture than if they have been exposed to a prime associated with a different culture.
*Hypothesis 2*: The magnitude of preference that a bicultural exhibits toward one culture over another culture will be influenced by the cultural prime.

It should be noted that while acculturation may involve the accumulation of cultural knowledge and facilitate the eventual identification with a given culture, (cf. Nguyen and Benet-Martínez, 2007), cultural priming will not have an effect on the level of acculturation in a given bicultural. Cultural priming activates the cultural knowledge and identity that has been developed as a result of acculturation (Hong et al., 2000), and therefore does not effect the level of acculturation itself.

## Methods

In order to determine whether biculturals could be primed to express loyalties toward different cultural groups, we designed an ostensibly low-stakes experimental study involving a soccer game. A sports game was chosen in order to avoid any sensationalism that may be associated with asking participants to respond to scenarios similar to the examples of discrimination discussed earlier, while still presenting the participants with two cultural groups engaging in competition. Sports psychology research has noted that individuals tend to root for athletes and sports teams that represent their identified in-group (Branscombe and Wann, 1992; Branscombe et al., 1993), a tendency that has been shown to be an expression of loyalty (Gwinner and Swanson, 2003). This design also served to mirror Branscombe and Wann's (1992) study, which used a boxing match between the United States and former Soviet Union from *Rocky IV* to assess participants' emotional reactions to two competing countries. A soccer game was selected for our study in order to avoid sports that may be strongly associated with a given culture (e.g., baseball with American culture or ping-pong with Chinese culture). The researchers were entirely self-funded for the entirety of this study. All experimental procedures were conducted in accordance with the relevant Institutional Review Board and in a manner congruent with the ethical guidelines of the American Psychological Association.

### Participant recruitment

The participants for this study were recruited from an undergraduate research participation pool of a university located in a large metropolitan city in the northeastern United States. The recruitment materials posted on the participant pool website requested self-identified Chinese-Americans who were fluent in Chinese-Mandarin to participate in a study on decision making.

A total of 188 participants signed up for the study. After filtering out incomplete responses, non-Chinese-American participants, and participants who did not finish the survey between 10 and 30 min, 136 responses remained. Incomplete responses were identified automatically by the online survey website. Non-Chinese-American participants were identified through a combination of self-identified ethnicity and the cultural background of both parents. Responses were filtered for response time as analysis of the response times showed wide variance, ranging from less than 1 min to over 60 h. This filter was utilized to ensure that participants did not proceed with the tasks too quickly, which would mean insufficient time viewing the stimulus materials, and also to ensure that participants did not take too much time and return to the study after a lengthy interruption, which would compromise the priming effect. Mean age was 21.69 years (*SD* = 3.07); 51.1% of the participants were female. Most of the participants were born overseas (73.68%), with 24.81% having been born in the United States, and 1 participant declined to disclose the country of birth. Of the participants born overseas, the average number of years spent in the United States was 10.68 years (*SD* = 6.85).

### Procedure

All participants completed study materials online, participating at times and locations of their convenience. The survey website was programmed in a manner that provided a controlled experimental environment in which participants were exposed to the cultural priming materials, described in more detail in the cultural priming section, for a set amount of time. The content and order of the study materials were presented to the participants as follows.

#### Cultural priming

**Figure 1:**
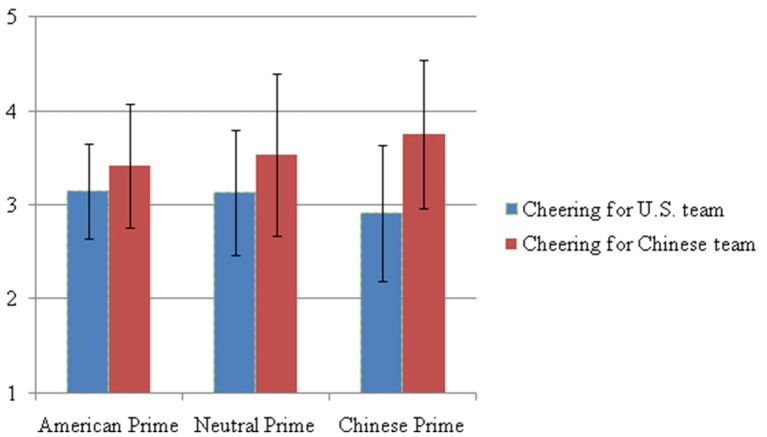
**Mean cheering and standard deviation for U.S. or Chinese team for each priming condition**.

Participants were randomly assigned to one of three cultural priming conditions: American, Chinese, and a Neutral priming condition for comparison. A combination of graphical and linguistic priming methods was used in this study in order to reinforce the cultural priming effect. The graphical priming materials were drawn from Mok's bicultural studies (Figure 1; Mok and Morris, 2012, 2013). Within each condition, each image was shown to participants for a minimum of 5 s before participants were allowed to proceed to the next image, in order to ensure that the participants properly processed the contents of each image. The minimum time was enforced by a timing function within the survey software. Given the possibility that language itself may serve as a prime (Ramirez-Esparza et al., 2006; Oyserman and Lee, 2008; LeChuga and Wiebe, 2009; Chen and Bond, 2010), in this study the cultural icons were paired with culture-congruent textual and audio languages. For the American priming condition all materials were presented in English text. For the Chinese priming condition, all materials were presented in Traditional Chinese-Mandarin text. For the intended neutral priming condition all materials were presented in English text due to the necessity of presenting participants with experiment text. Given the general environment surrounding the study, English was selected as the default language to use.

#### Scenario

Following the cultural prime, participants were informed they would be shown a simulation of a soccer game and be asked to answer a few questions about the audio commentary that had accompanied the game and the game itself. The scenario was adapted from the methodology used in Branscombe and Wann's (1992) study. We presented each participant with a short 5-min video clip of a soccer game involving an American team and a Chinese team. The video clip was compiled from recordings of a simulated soccer match between the United States and China from FIFA 11, a soccer video game. The soccer match was run as an automated simulation between the two teams, utilizing the video game's artificial intelligence (AI) with no input from the researchers. The resulting video clip consisted of passes between the AI players of the respective countries, interceptions, and attempts at goal shots; however, no scoring shots were included in the clip in order to provide the perception of an inconclusive game. The cultural priming was further reinforced through the usage of voiceover commentary for the video clip recorded by native speakers of each language. Both commentators read from a pre-written script that described the same actions displayed in the video clip. Although all participants were shown the exact same video graphics, the audio commentary that accompanied the video clip differed between priming conditions. For the American priming condition the audio commentary was in English, while for the Chinese priming condition the audio commentary was in Chinese-Mandarin. For the Neutral condition, no audio commentary was provided.

#### Expression of loyalty

Following the soccer game, we assessed participants' expression of loyalty for each of the teams engaged in the simulated match by measuring their rooting behaviors. Due to the possibility that participants may root for both teams despite the cultural priming, we assessed rooting behavior for the participants toward both teams. Participants were asked to indicate to what extent they would root for the American and Chinese teams. This was measured by two items: “I would cheer for the US team,” to assess rooting behavior for the American team, and “I would cheer for the Chinese team,” to assess rooting behavior for the Chinese team. Participants responded on a 5-point Likert scale, with 1 denoting “Extremely Disagree” and 5 denoting “Extremely Agree.” The two items were presented in randomized order.

#### Behavioral acculturation

To assess participants' levels of acculturation with American and Chinese cultures, Tsai et al. (2000) General Ethnicity Questionnaire (GEQ) was adopted for this study. The GEQ is a 39-item, 5-point Likert scale (1 = Strongly Disagree, 5 = Strongly Agree) measure of acculturation that can be tailored to specific cultures, with items such as “I listen to American [Chinese] music,” and “I celebrate American [Chinese] holidays” (GEQ-American, α = 0.87, GEQ-Chinese, α = 0.85 in the present sample). All participants completed two versions of the GEQ: American and Chinese, presented in randomized order. The acculturation measures were presented after the presentation of the main independent and dependent variables so as to not inadvertently prime the participants. Analysis afterwards showed no significant differences in acculturation between the two cultural priming conditions, indicating that, as expected, results obtained from the acculturation measures were not influenced by the cultural primes.

#### Demographics and background variables

At the conclusion of the study, participants were asked to complete a demographic questionnaire, including items about their cultural backgrounds and their parents' country of origin. Participants were also asked to provide information on the types of sports they typically watched, whether they were familiar with soccer, and whether they supported any particular soccer teams.

## Results

Participants in this study were overall more acculturated with Chinese culture (*M* = 3.78, *SD* = 0.43) than American culture (*M* = 3.25, *SD* = 0.42), *t*_(136)_ = −10.31, *p* < 0.05, Cohen's d = −0.88, in line with past studies on biculturals that have also shown similar acculturation patterns (Tsai et al., 2000; Benet-Martínez et al., 2002; Benet-Martínez and Haritatos, 2005; Cheng et al., 2006; Miramontez et al., 2008; Zou et al., 2008). Therefore, despite the differences found in the levels of acculturation in American and Chinese cultures, the sample was bicultural due to being acculturated with both cultures to some extent. The correlations for the variables investigated, along with the means and standard deviations for each, are presented in Table 1.

**Table 1:** **Observed correlations, means, and standard deviations of study variables**.

	***M***	***SD***	**1**	**2**	**3**	**4**	**5**	**6**
Age	21.69	3.07						
Sex			**−0.22**					
Prime			−0.05	−0.11				
American acculturation	3.25	0.42	−0.11	0.01	0.01			
Chinese acculturation	3.78	0.43	−0.04	−0.07	0.03	0.02		
Cheering for U.S. team	3.02	0.67	0.12	0.14	**−0.16**	0.10	−0.11	
Cheering for Chinese team	3.61	0.79	0.06	−0.05	**0.18**	−0.12	**0.41**	−0.12

*N = 136. p < 0.05 for values in bold*.

Participants were overall not very familiar with soccer (*M* = 1.99 on a 5-point Likert scale, *SD* = 0.90, with “2” on the scale being “A little.”), and out of those that expressed support for specific soccer teams, none indicated support for American or Chinese soccer teams. There were also no significant differences between sex in terms of familiarity with soccer. Therefore, no participants were filtered out due to preconceived loyalties toward the soccer teams from either country.

Given the 3 (between subjects priming condition: American, Neutral, and Chinese) × 2 (within subjects repeated measures: Cheering for US team, and Cheering for Chinese team), we conducted a mixed ANOVA analysis. Analyses conducted while controlling for American and Chinese acculturation, sex, and familiarity with soccer did not change the significance or direction of the results, therefore results from analyses procedures most consistent with established methodology found in literature, controlling for acculturation and familiarity with soccer, are reported. Additionally, given discussions on the common usage of demographics characteristics as a proxy for acculturation (cf. Nguyen and Benet-Martínez, 2007), we did not use country of birth as a control variable, and instead used level of acculturation. Initial results showed a significant Prime × Cheering interaction, *F*_(2, 130)_ = 4.89, *p* = 0.01, $\eta_{\text{p}}^{2}$ = 0.07, indicating that the cultural priming did have an effect on the cheering. In order to better understand the results, we further examined the simple main effects. Univariate analysis on whether the cultural priming changed the level cheering for each team demonstrated small effects ($\eta_{\text{p}}^{2}$ = 0.04 for both cheering outcomes; Cohen, 1988); however, these results were not statistically significant. Therefore, Hypothesis 1 is not supported. However, multivariate analysis comparing what effects the cultural priming had on the differences *between* the two cheering measures showed significant differences. Results showed that the cultural priming increased the magnitude of the preference participants displayed toward the Chinese team over the American team. Specifically, while the participants did not express significantly different cheering for either team under the American priming condition, the participants did express significantly lower levels of cheering for the American team (*M* = 3.13, *SD* = 0.67) compared to the Chinese team (*M* = 3.53, *SD* = 0.86) under the Neutral priming condition, *F*_(1, 130)_ = 6.10, *p* = 0.02, $\eta_{\text{p}}^{2}$ = 0.05. This difference was magnified under the Chinese priming condition, *F*_(1, 130)_ = 48.29, *p* = 0.00, $\eta_{\text{p}}^{2}$ = 0.28, with the participants expressing significantly lower level of cheering for the American team (*M* = 2.91, *SD* = 0.72) compared to the Chinese team (*M* = 3.75, *SD* = 0.79). Therefore, Hypothesis 2 is supported. Table 2 presents the means and standard deviations for the dependent variables under all priming conditions, and Figure 1 presents the results graphically. Implications of the findings are discussed in the following section.

**Table 2:** **Mean rooting behavior by priming condition**.

	**American prime** ***n*_1_ = 33**		**Neutral prime** ***n*_2_ = 38**		**Chinese prime** ***n*_3_ = 65**	
	***M***	***SD***	***M***	***SD***	***M***	***SD***
Cheering for the U.S. team	3.15	0.51	3.13	0.67	2.91	0.72
Cheering for the Chinese team	3.42	0.66	3.53	0.86	3.75	0.79

## Discussion and implications

The results in this study partially supported the proposed hypotheses in that the different priming conditions did not influence how the Chinese-American participants in this study rooted for each of the soccer teams, Chinese or U.S., however the different priming conditions did influence the difference by which the Chinese-American participants in this study cheered for the Chinese over the U.S. soccer teams. These findings indicate that the degree to which biculturals express loyalty for each of the respective cultures when they are in direct competition with each other can be externally influenced by priming. This shows that bicultural loyalties can be malleable to a certain degree.

Most notably, cultural primes did have an effect on the magnitude of the difference by which biculturals express loyalty toward their host vs. the home culture. When their host cultural identity is activated, biculturals do not differ in their expressed loyalty, while the activation of the home identity causes biculturals to express the highest degrees of loyalty toward their home culture compared to the host culture, while under a supposedly culturally neutral situation in which no specific identity is being activated, their expression of home loyalty is slightly reduced. Essentially, biculturals do not feel differential loyalties when their host cultural identity is primed. However, when their host cultural identity is not activated, then their loyalty for the home culture becomes significantly higher.

Returning to societal implications, our findings suggest a Chinese-American living in the United States and exposed to American culture and English in daily life may not feel large disparities in terms of loyalties toward China or the United States, although they would be slightly more loyal to China. However, when Chinese cultural elements are present in the environment and thereby activating the Chinese identity, then the individual would experience heightened loyalties toward China. It is not exactly clear why this is the case. It is possible because our participants largely originated from China, they are innately loyalty to the Chinese team over the U.S. team regardless of the cultural prime. The primary effect cultural primes have on this loyalty is the degree to which they would express loyalty toward one team over another. In contrast, biculturals born in the United States may innately support the U.S. team over the Chinese team. The likely explanation is that the participants, due to exhibiting higher levels of Chinese acculturation than American acculturation, also had higher levels of cultural identification with Chinese culture than American culture even though this identification can still be manipulated by cultural primes. However, due to the limited sample size of the current study we were not able to conduct these *post-hoc* analyses. Future research may investigate this phenomenon further and also explore whether differences in behavioral acculturation, which were used as control variables in the current study, and cultural identification may have an impact on the expression of loyalty.

There may also be other possible moderators in this phenomenon. First and foremost is bicultural identity integration (BII). Research on BII show that how cultural stimuli activate cultural knowledge and identities in biculturals can be moderated by whether biculturals view their dual cultural identities as being conflicting and distant from each other (Benet-Martínez et al., 2002). A bicultural who feels more conflicted, or Low BII, responds contrastively to cultural stimulus, e.g., an American cultural prime activates the Asian knowledge frame and identity, while a bicultural who feels less conflicted, or High BII, responds congruently to cultural stimulus, e.g., an American cultural prime activates the American knowledge frame and identity. In the context of this study, a Low BII bicultural might cheer more for the Chinese team when exposed to an American prime, while a High BII bicultural might cheer more for the American team when exposed to an American prime.

Another possible moderator is that of in-group threat. Specifically, out-group derogation is a result of threat to the in-group, with individuals derogating lower-status groups when faced with a threat to the in-group, a concept known as downward compensation (Wills, 1981). In the context of this study it is possible that a Chinese-American bicultural would derogate against a team from a culture perceived as being lower-status when the Chinese team is being threatened. However, the current study design, which focuses on cheering for a given team, would need to be adjusted to reflect that individuals could also react negatively toward a competing team. This study design would be particularly interesting given findings that suggest intergroup bias is driven more by love for the in-group rather than derogation toward the out-group (Halevy et al., 2008, 2012), suggesting that downward compensation is engaged in order to make the in-group easier to love.

Another possible moderator is that of personal or collective discrimination. Specifically, research suggests that individuals who belong to lower status groups exhibit higher degrees of intergroup bias (Leonardelli and Brewer, 2001). Therefore, in the context of the current study, depending on the status differential between the two competing sports teams, there may be a larger differential between cheering for the in-group team than the out-group team.

Although bicultural research has progressed rapidly in recent years, many questions regarding bicultural cognition, identity, and social identity remain to be answered. The results obtained from this study lend one more piece toward the puzzle of bicultural social identity and how biculturals express their loyalty toward different cultural groups, while highlighting several factors that still merit further research.

## Ethics statement

This study was approved by the institutional review boards of Baruch College/CUNY and SUNY Farmingdale State College and found to be exempt per 45 CFR 46.101(b)(2). Participants were presented with an electronic version of the consent form. Participants read the consent form and indicated through check boxes whether they consented. Those who consented continued with the study, while those who did not consent were thanked for their time and did not continue with the study. Undergraduate students were involved in this study. Students who chose not to participate in the research were allowed to do so at any time during their participation. Students were recruited via a departmental Research Participant Pool. Students in the Research Pool may choose to fulfill their research credit requirements through two methods: participation in research studies, or writing short papers. Participation in research is not a requirement, and withdrawal from research studies in no way harms the student's grades or standing.

## Author contributions

AC contributions to manuscript: Literature review, initial hypothesis development, study design, data collection, data analysis, and writing BM contributions to manuscript: Hypothesis development, study design, data collection, data analysis, writing, and editing.

### Conflict of interest statement

The authors declare that the research was conducted in the absence of any commercial or financial relationships that could be construed as a potential conflict of interest.

## References

[B1] Asher-Shapiro A (2015). Why Does the FBI Keep Arresting Asian-American Scientists? VICE News. Available online at: https://news.vice.com/article/why-does-the-fbi-keep-arresting-asian-american-scientists

[B2] Benet-Martínez V. Haritatos J. (2005). Bicultural Identity Integration (BII): components and psychosocial antecedents. J. Pers. 73, 1015–1050. 10.1111/j.1467-6494.2005.00337.x 15958143

[B3] Benet-Martínez V. Leu J. Lee F. Morris M. W. (2002). Negotiating biculturalism: cultural frame switching in biculturals. J. Cross Cult. Psychol. 33, 492–516. 10.1177/0022022102033005005

[B4] Berry J. W. Phinney J. S. Sam D. L. Vedder P. (2006). Immigrant youth: acculturation, identity, and adaptation. Appl. Psychol. 55, 303–332. 10.1111/j.1464-0597.2006.00256.x

[B5] Berry J. W. Sam D. (1997). Acculturation and adaptation, in Handbook of Cross-Cultural Psychology, Vol. 3, eds Berry J. W. Segall M. Kagitcibasi C.(Boston: Allyn & Bacon), 291–326.

[B6] Bond M. H. (ed.). (1993). Between the Yin and the Yang: The Identity of the Hong Kong Chinese. Hong Kong: Hong Kong Chinese University.

[B7] Bond M. H. Cheung T.-S. (1983). College students' spontaneous self-concept: the effect of culture among respondents' in Hong Kong, Japan, and the United States. J. Cross Cult. Psychol. 14, 153–171. 10.1177/0022002183014002002

[B8] Brannen M. Y. Thomas D. C. (2010). Bicultural individuals in organizations: implications and opportunity. Int. J. Cross Cult. Manag. 10, 5–16. 10.1177/1470595809359580

[B9] Branscombe N. R. Wann D. L. (1992). Physiological arousal and reactions to outgroup members during competitions that implicate an important social identity. Aggress. Behav. 18, 85–93. 10.1002/1098-2337(1992)18:2<85::AID-AB2480180202>3.0.CO;2-9

[B10] Branscombe N. R. Wann D. L. Noel J. G. Coleman J. (1993). In-group or out-group extremity: importance of the threatened social identity. Pers. Soc. Psychol. Bull. 19, 381–388. 10.1177/014616729319400326621267

[B11] Brewer M. B. (1999). Multiple identities and identity transition: implications for Hong Kong. Int. J. Intercult. Relat. 23, 187–197. 10.1016/S0147-1767(98)00034-0

[B12] Brewer M. B. Ho H.-K. Lee J.-Y. Miller N. (1987). Social identity and social distance among Hong Kong schoolchildren. Pers. Soc. Psychol. Bull. 13, 156–165. 10.1177/0146167287132002

[B13] Chen S. X. Bond M. H. (2010). Two languages, two personalities? Examining language effects on the expression of personality in a bilingual context. Pers. Soc. Psychol. Bull. 36, 1514–1528. 10.1177/0146167210385360 20944020

[B14] Cheng C.-Y. Lee F. Benet-Martínez V. (2006). Assimilation and contrast effects in cultural frame switching: bicultural identity integration and valence of cultural cues. J. Cross Cult. Psychol. 37, 742–760. 10.1177/0022022106292081

[B15] Cohen J. (1988). Statistical Power Analysis for the Behavioral Sciences (2nd Edn.). Hillsdale, NJ: Erlbaum.

[B16] Devos T. (2006). Implicit bicultural identity among Mexican American and Asian American college students. Cult. Divers. Ethn. Minor. Psychol. 12, 381–402. 10.1037/1099-9809.12.3.381 16881745

[B17] Gwinner K. Swanson S. R. (2003). A model of fan identification: antecedents and sponsorship. J. Serv. Market. 17, 275–294. 10.1108/08876040310474828

[B18] Halevy N. Bornstein G. Sagiv L. (2008). “In-group love” and “out-group hate” as motives for individual participation in intergroup conflict: a new game paradigm. Psychol. Sci. 19, 405–411. 10.1111/j.1467-9280.2008.02100.x 18399895

[B19] Halevy N. Weisel O. Bornstein G. (2012). “In-group love” and “out-group hate” in repeated interaction between groups. J. Behav. Decis. Mak. 25, 188–195. 10.1002/bdm.726

[B20] Hewstone M. Islam M. R. Judd C. M. (1993). Models of crossed categorization and intergroup relations. J. Pers. Soc. Psychol. 64, 779–793. 10.1037/0022-3514.64.5.779 8505707

[B21] Hogg M. A. Terry D. J. White K. M. (1995). A tale of two theories: a critical comparison of identity theory with social identity theory. Soc. Psychol. Q. 58, 255–269. 10.2307/2787127

[B22] Hong Y.-Y. Morris M. W. Chiu C. Benet-Martínez V. (2000). Multicultural minds: a dynamic constructivist approach to culture and cognition. Am. Psychol. 55, 709–720. 10.1037/0003-066X.55.7.709 10916861

[B23] Kang S. K. Bodenhausen G. V. (2015). Multiple identities in social perception and interaction: challenges and opportunities. Ann. Rev. Psychol. 66, 547–574. 10.1146/annurev-psych-010814-015025 25061671

[B24] Krieg G. (2015). Muslim Americans: Current Political Climate Worse than after 9/11. CNN. Available online at: http://edition.cnn.com/2015/11/20/politics/paris-attacks-trump-carson-bush-muslims-refugees-mosques

[B25] LaFromboise T. Coleman H. L. K. Gerton J. (1993). Psychological impact of biculturalism: evidence and theory. Psychol. Bull. 114, 395–412. 10.1037/0033-2909.114.3.395 8272463

[B26] LeBoeuf R. A. Shafir E. Bayuk J. B. (2010). The conflicting choices of alternating selves. Organ. Behav. Hum. Decis. Process 111, 48–61. 10.1016/j.obhdp.2009.08.004

[B27] LeChuga J. Wiebe J. S. (2009). Can language prime cultural in Hispanics? The differential impact of self-construals in predicting intention to use a condom. Int. J. Psychol. 44, 468–476. 10.1080/00207590902835710 22029664 PMC3415329

[B28] Leonardelli G. J. Brewer M. B. (2001). Minority and majority discrimination: when and why. J. Eur. Soc. Psychol. 37, 468–485. 10.1006/jesp.2001.1475

[B29] Liebkind K. (2003). Acculturation, in Blackwell Handbook of Social Psychology: Intergroup Processes, eds Brown R. Gaertner S.(Malden, MA: Blackwell), 386–408.

[B30] Liu S. (2015). Searching for a sense of place: identity negotiation of Chinese immigrants. Int. J. Intercult. Relat. 46, 26–35. 10.1016/j.ijintrel.2015.03.020

[B31] Miramontez D. R. Benet-Martínez V. Nguyen A.-M. D. (2008). Bicultural identity integration and self/group personality perceptions. Self Identity 7, 430–445. 10.1080/15298860701833119

[B32] Mok A. Cheng C.-Y. Morris M. W. (2010). Matching versus mismatching cultural norms in performance appraisal: effects of the cultural setting and bicultural identity integration. Int. J. Cross Cult. Manag. 10, 17–35. 10.1177/1470595809359584

[B33] Mok A. Morris M. W. (2009). Cultural chameleons and iconoclasts: assimilation and reactance to cultural cues in biculturals' expressed personalities as a function of identity conflict. J. Exp. Soc. Psychol. 45, 884–889. 10.1016/j.jesp.2009.04.004

[B34] Mok A. Morris M. W. (2010). Asian-Americans' creative styles in Asian and American situations: assimilative and contrastive responses as a function of bicultural identity integration. Manag. Organ. Rev. 6, 371–390. 10.1111/j.1740-8784.2010.00190.x

[B35] Mok A. Morris M. W. (2012). Attentional focus and the dynamics of dual identity integration: evidence from Asian Americans and female lawyers. Soc. Psychol. Pers. Sci. 3, 597–604. 10.1177/1948550611432769

[B36] Mok A. Morris M. W. (2013). Bicultural self-defense in consumer contexts: self-protection motives are the basis for contrast versus assimilation to cultural cues. J. Consum. Psychol. 23, 175–188. 10.1016/j.jcps.2012.06.002

[B37] Ng S. H. Lai J. C. L. (2009). Effects of culture priming on the social connectedness of the bicultural self. J. Cross Cult. Psychol. 40, 170–186. 10.1177/0022022108328818

[B38] Ng S. H. Lai J. C. L. (2011). Bicultural self, multiple social identities, and dual patriotisms among ethnic Chinese in Hong Kong. J. Cross Cult. Psychol. 42, 89–103. 10.1177/0022022110361715

[B39] Nguyen A.-M. D. Benet-Martínez V. (2007). Biculturalism unpacked: components, measurement, individual differences, and outcomes. Soc. Pers. Psychol. Compass 1, 101–114. 10.1111/j.1751-9004.2007.00029.x

[B40] Nguyen A.-M. D. Benet-Martínez V. (2013). Biculturalism and adjustment: a meta-analysis. J. Cross Cult. Psychol. 44, 122–159. 10.1177/0022022111435097

[B41] Oyserman D. Lee S. W. S. (2008). Does culture influence what and how we think? Effects of priming individualism and collectivism. Psychol. Bull. 134, 311–342. 10.1037/0033-2909.134.2.311 18298274

[B42] Phinney J. S. Horenczyk G. Liebkind K. Vedder P. (2001). Ethnic identity, immigration, and well-being: an interactional perspective. J. Soc. Issues 57, 493–510. 10.1111/0022-4537.00225

[B43] Pilar J. A. D. Udasco J. O. (2004). Marginality theory: the lack of construct validity. Hisp. J. Behav. Sci. 26, 3–15. 10.1177/0739986303261813

[B44] Purdy M (2001). The Making of a Suspect: The Case of Wen Ho Lee. The New York Times. Available online at: http://www.nytimes.com/2001/02/04/us/the-making-of-a-suspect-the-case-of-wen-ho-lee.html?pagewanted=all

[B45] Ramirez-Esparza N. Gosling S. D. Benet-Martínez V. Potter J. P. Pennebaker J. W. (2006). Do bilinguals have two personalities? A special case of cultural frame switching. J. Res. Pers. 40, 99–120. 10.1016/j.jrp.2004.09.001

[B46] Roccas S. Brewer M. B. (2002). Social identity complexity. Pers. Soc. Psychol. Rev. 6, 88–106. 10.1207/S15327957PSPR0602_01

[B47] Stewart P. (2016). U.S. Navy Officer Suspected of Passing Secrets to Taiwan, China. Washington, DC: Reuters.

[B48] Tajfel H. Turner J. (1979). An integrative theory of intergroup conflict, in The Social Psychology of Intergroup Relations, eds Austin W. Worchel S.(Monterey, CA: Brooks/cole), 33–47.

[B49] Tsai J. L. Ying Y.-W. Lee P. A. (2000). The meaning of “being Chinese” and “being American”: variation among Chinese American young adults. J. Cross Cult. Psychol. 31, 302–332. 10.1177/0022022100031003002

[B50] Turner J. C. Hogg M. A. Oakes P. J. Reicher S. D. Wetherell M. S. (1987). Rediscovering the Social Group: A self-categorization Theory. New York, NY: Blackwell.

[B51] Van Vugt M. Hart C. M. (2004). Social identity as social glue: the origins of group loyalty. J. Pers. Soc. Psychol. 86, 585–598. 10.1037/0022-3514.86.4.585 15053707

[B52] Verkuyten M. Pouliasi K. (2006). Biculturalism and group identification: the mediating role of identification in cultural frame switching. J. Cross Cult. Psychol. 37, 312–326. 10.1177/0022022106286926

[B53] Wills T. A. (1981). Downward comparison principles in social psychology. Psychol. Bull. 90, 245–271. 10.1037/0033-2909.90.2.245

[B54] Zou X. Morris M. W. Benet-Martínez V. (2008). Identity motives and cultural priming: cultural (dis) identification in assimilative and contrastive responses. J. Exp. Soc. Psychol. 44, 1151–1159. 10.1016/j.jesp.2008.02.001

